# Heat Stress Impairs Maternal Endometrial Integrity and Results in Embryo Implantation Failure by Regulating Transport-Related Gene Expression in Tongcheng Pigs

**DOI:** 10.3390/biom12030388

**Published:** 2022-03-02

**Authors:** Weisi Lian, Dengying Gao, Cheng Huang, Qiqi Zhong, Renwu Hua, Minggang Lei

**Affiliations:** 1 Key Laboratory of Agricultural Animal Genetics, Breeding, and Reproduction of the Ministry of Education, Key Laboratory of Swine Genetics and Breeding of the Ministry of Agriculture, College of Animal Science and Technology, Huazhong Agricultural University, Wuhan 430070, China; lianweisi2021@163.com (W.L.); gdyy0107@163.com (D.G.); hc18283901239@163.com (C.H.); zhongqiqihamster@163.com (Q.Z.); huarenwu1994@163.com (R.H.); 2 National Engineering Research Center for Livestock, Huazhong Agricultural University, Wuhan 430070, China; 3 Department of Pig Production, The Cooperative Innovation Center for Sustainable Pig Production, Wuhan 430070, China

**Keywords:** heat stress, porcine endometrial cells, tight junction, cell adhesion, transport activity, *S100A9*

## Abstract

Heat stress (HS) poses a significant threat to production and survival in the global swine industry. However, the molecular regulatory effects of heat stress on maternal endometrial cells are poorly understood in pigs during early embryo implantation. In this study, we systematically examined morphological changes in the endometrium and the corresponding regulation mechanism in response to HS by combining scanning electron microscopy (SEM), hematoxylin/eosin (H&E) staining, western blot, and RNA-seq analyses. Our results showed that HS led to porcine endometrium damage and endometrial thinness during embryo implantation. The expression levels of cell adhesion-related proteins, including N-cadherin and E-cadherin, in the uterus were significantly lower in the heat stress group (39 ± 1 °C, *n* = 3) than in the control group (28 ± 1 °C, *n* = 3). A total of 338 up-regulated genes and 378 down-regulated genes were identified in porcine endometrium under HS. The down-regulated genes were found to be mainly enriched in the pathways related to the microtubule complex, immune system process, and metalloendopeptidase activity, whereas the up-regulated genes were mainly involved in calcium ion binding, the extracellular region, and molecular function regulation. *S100A9* was found to be one of the most significant differentially expressed genes (DEGs) in the endometrium under HS, and this gene could promote proliferation of endometrial cells and inhibit their apoptosis. Meanwhile, HS caused endometrial epithelial cell (EEC) damage and inhibited its proliferation. Overall, our results demonstrated that HS induced uterine morphological change and tissue damage by regulating the expression of genes associated with calcium ions and amino acid transport. These findings may provide novel molecular insights into endometrial damage under HS during embryo implantation.

## 1. Introduction

Embryo implantation is a structural connection between an embryo and the maternal endometrium to allow material exchange, and it plays a crucial role in female reproduction [1,2]. Endometrium receptivity is the basis for successful embryo implantation and is influenced by various stresses [3,4,5,6]. At present, extreme heat in summer has become more frequent due to global climate warming and it has a more severe impact on livestock and poultry [7,8,9]. Heat stress (HS) is a non-specific physiological reaction of an organism in response to thermal environmental stimuli [10]. Since pigs lack sweat glands and have thicker subcutaneous fat, they cannot dissipate heat through the evaporation process, making them more susceptible to high temperatures than other animals [11,12]. HS can adversely affect pig growth and development, reproduction performance, and meat quality, thereby causing significant economic losses in the swine industry [13,14,15,16]. The Tongcheng pig is a Chinese indigenous breed, and it is mainly distributed in Hubei province. Tongcheng pigs have characteristics of early sexual maturity, prolific reproduction, strong stress resistance, and delicious meat [17]. In recent years, the summer temperatures in the southern region of China have gradually increased, with the highest temperature in some places even exceeding 40 °C and lasting for a long time. The occurrence of HS has great impacts on pregnancy and fetal health; thus, the potential risks of HS arouse wide concern.

Previous studies have revealed that HS leads to a small corpus luteum and early embryonic death [18,19]. HS alters hormone secretion, induces autophagy, and increases anti-apoptotic signaling in pig ovaries during follicular development [20,21]. Successful embryo implantation is dependent on a receptive endometrium, functional blastocyst, and the synchronization between them. During early implantation, embryos are susceptible to embryo resorption and miscarriage [22]. Appropriate endometrial thickness is essential for embryo implantation and pregnancy maintenance. Endometrial morphology and thickness have been confirmed to be associated with uterine receptivity [23,24,25]. In addition, the development of endometrial epithelial cells is the “switch” that initiates embryo adhesion. The essential functions of epithelial cells are to provide a barrier and to transport directional materials. The uterine epithelium accepts an embryo implantation only during the window period, but it is a barrier against blastocyst implantation outside of this window [26,27,28]. It has been reported that a uterus can form microvillus structures that participate in cattle gestation, during which, the uterus undergoes complex changes, such as the expression change of some adhesion molecules to ensure proper implantation of the embryo into the uterus. Previous Western blot and cytoskeleton immunostaining analyses have showed that occludin and claudin1 proteins are changed during porcine early embryo implantation [29,30]. Cadherins comprised of a large family of cell adhesion molecules are key regulators for cell adhesion, sorting, and invasion. When the expression of cadherins is reduced, the uterus is not in a receptive state and fails to provide the proper embryo implantation environment [31]. Although it is well acknowledged that the uterine microenvironment is a vital prerequisite for successful embryo attachment, the effects of HS on the uterus and the related molecular mechanisms remain largely unknown.

Considering this, this study aimed to use RNA-seq in combination with the examination of endometrium structural changes to assess the effect of HS on uterine and potential molecular mechanisms in Tongcheng pigs. Our data indicated that HS might alter the integrity of the uterus, which was not conducive to female reproductive maintenance. By detecting tight junction-related proteins we determined the regulatory effect of HS on endometrium integrity and thickness during female reproduction. Combined analyses of RNA-seq, H&E, and SEM showed that maternal HS affected the expression of genes related to cell adhesion and uterus ion transport capacity during implantation, thus disrupting embryo adhesion to the endometrium, which would eventually result in implantation and pregnancy failure.

## 2. Materials and Methods

### 2.1. Sample Collection

Six female Tongcheng pigs with similar ages and genetic backgrounds (100 days old, 45 ± 5 kg) were selected and randomly divided into a control group and a heat stress group. These experimental pigs were housed in the Jingpin farm and slaughtered in the slaughterhouse in Huazhong Agricultural University (Wuhan, China). Every gilt underwent a warm shower to relax the pig prior to slaughter. Then they were stunned with a low-voltage electric shock to reduce the pain and were exsanguinated to death by puncturing the carotid artery. All the animal experiments were reviewed and approved by the Institutional Animal Care and Use Committee in Huazhong Agricultural University, and the principles of laboratory animal care were followed.

In the third estrus, the gilts were subjected to artificial insemination and the end of the second insemination was day 0 of pregnancy. The gilts were then randomly divided into two groups at day 11 post implantation: a control group (28 ± 1 °C, *n* = 3) and a heat-stressed group (39 ± 1 °C, *n* = 3). HS lasted four days (from 9:00 am to 16:00 pm). The pigs in the control group and the heat stress group were kept separately. All pigs had free access to water and were fed twice a day. Uteri were harvested from slaughtered pigs on day 14 of pregnancy. The conceptuses were flushed from each uterine horn with sterile phosphate buffered saline (PBS, 0.1 M, Ph 7.4) and subsequently opened longitudinally on the inner side. The formation of the conceptus on day 14 of pregnancy was filamentous. Endometrial samples were collected and stored at −80 °C for further analyses.

### 2.2. Electron Microscopy Observation

The endometrial tissues were immediately removed and cut into 3 mm^2^ small cubes and washed with PBS. Afterwards, the samples were first fixed with 2.5% glutaraldehyde at room temperature for 2 h, then they were stored at 4 °C for 24 h, rinsed with the same PBS, fixed with 1% buffered osmium tetroxide, and rewashed three times with the PBS. The samples were dehydrated in ethanol and permeated with propylene oxide-araldite compound before they were embedded in araldite. The resultant samples were subjected to scanning electron microscopy (SEM) with a HITACHI Regulus 8100 (Hitachi, Tokyo, Japan) scanning electron microscope at 6.0 kV.

### 2.3. Histological Analysis

For the histological analysis, uterine samples were fixed in 4% paraformaldehyde solution for 24 h, followed by paraffin embedding and sectioning at 4 μm. The sections were stained with hematoxylin and eosin (H&E) according to the standard protocol. Histological observations were performed under an Olympus microscope (BX53, Olympus, Tokyo, Japan) with the Panoramic Viewer system (1.15.3).

### 2.4. Western Blot

The total protein was extracted from uterine tissues with RIPA lysis buffer added with protease inhibitors. All samples were centrifuged at 12,000 r/min for 10 min at 4 °C, and the supernatants were collected. Protein concentrations were quantified with the Pierce^®^ BCA Protein Assay Kit (Thermo Fisher Scientific, Waltham, MA, USA). Proteins were separated with 10% SDS/PAGE gels and transferred to PVDF membranes. The membranes were incubated with the following primary antibodies against E-cadherin (20874-1-AP, Proteintech, Wuhan, China), N-cadherin (66219-1-lg, Proteintech, Wuhan, China), occludin (27260-1-AP, Proteintech, Wuhan, China), claudin1 (kindly provided by Jianwei Gao), and GAPDH (60004-1-lg, Proteintech, Wuhan, China), and then incubated with HRP-conjugated secondary antibodies. Subsequently, the membranes were incubated with developing solution (1705060, Bio-rad, Hercules, CA, USA). A Western blot assay was conducted with GAPDH as an internal reference.

### 2.5. RNA Extraction and Sequencing

The total RNA was extracted from the six samples with TRIzol reagent (Invitrogen, Carlsbad, CA, USA) according to the manufacturer’s instructions. The RNA concentration, purity, and integrity were determined with a NanoPhotometer spectrophotometer (Thermo Scientific, Waltham, MA, USA) and the RNA Nano 600 Assay Kit on the Bioanalyzer 2100 system (Agilent Technologies, Santa Clara, CA, USA). The library was constructed using the NEBNext UltraTM RNA Library Prep Kit for Illumina (NEB, Beijing, China) following the manufacturer’s recommendations.

### 2.6. GO and KEGG Enrichment Analysis

Differentially expressed genes (DEGs) (*p*-value < 0.05) were identified. Gene ontology (GO) and the Kyoto Encyclopedia of Genes and Genome (KEGG) enrichment analyses were preformed to determine the biological processes and molecular functions that the DEGs were involved in by using the clusterProfiler R package.

### 2.7. Protein–Protein Interaction Network Analysis (STRING Analysis)

The STRING online database (version 11: https://string-db.org/, 21 August 2020) was first applied to obtain the PPI information of the differentially expressed transcripts. The interaction network of differentially expressed transcripts was established with a minimal confidence score of 0.4.

### 2.8. RT-qPCR

We chose 10 genes to verify the accuracy of the RNA-seq results using RT-PCR. The cDNA was synthesized with the PrimeScript reverse transcriptase reagent kit (TaKaRa, Dalian, China). The cDNA was amplified using a SYBR^®^ Green PCR Master Mix (Bimake, Houston, TX, USA) on a LightCycler 480II quantitative real-time PCR (qRT-PCR). The gene relative expression levels were normalized to the expression level of β-Actin by using the 2(−ΔΔCt) method.

### 2.9. Cell Preparation and Culture Conditions

Primary porcine endometrial epithelial cells (EECs) were used in this study. Isolation and culture were performed by previously reported methods with minor modifications [32]. In a sterile environment, the uterine tissues were cut into small pieces (1 mm^3^), transferred to collagenase I (Gibco, Grand Island, NY, USA) solution, digested at 37 °C for 2.5 h, and transferred to complete medium. Porcine endometrial cells were cultured in Dulbecco’s modified Eagle’s medium/F-12 (DMEM/F12) and supplemented with 10% fetal bovine serum (FBS, Gibco) and 1% penicillin-streptomycin (Gibco) at 37 °C under 5% CO_2_ in humidified air. Porcine sequence-coding *S100A9* fragment (GenBank accession no. NM_001177906.1) was cloned into the mammalian expression vector pcDNA3.1 to construct a recombinant plasmid, which was designated as pcDNA3.1-*S100A9*. We used lipofectamine 2000 (Invitrogen, Waltham, MA, USA) for siRNA transfection as previously described. The used primer pair included F: GCT CTA GAG CTC CTG GGC TTG GAC AGA GT and R: GGG GTA CCC CAA GGT GGA CAG GGG TGC AT for the overexpression of *S100A9*. The sense strand sequence and antisense strand sequence of *S100A9* were 5′-CCC UGA ACC AGA AAG AAU UTT-3′ and 5′-AAU UCU UUC UGG UUC AGG GTT-3′, respectively, with sense strand sequence 5′-UUC UCC GAA CGU GUC ACG UTT-3′ and antisense strand sequence 5′-ACG UGA CAC GUU CGG AGA ATT-3′ used as a negative control (NC).

### 2.10. Cell Cycle Analysis

The effect of *S100A9* on the cell cycle distribution was analyzed using cell flow cytometry. After transfection with pcDNA3.1(+)-*S100A9*, pcDNA3.1(+), si-*S100A9*, and NC, the cells were incubated for 48 h, trypsinized, washed with cold PBS, and fixed in 75% ethanol overnight at 4 °C. After fixation, the cells were rewashed and then re-suspended in 1 mL of propidium iodide (PI) staining reagent containing 50 mg ml-1 PI and 100 g ml-1 DNase-free RNase A, followed by incubation for 30 min at 4 °C in the dark before cell cycle analysis. The cell cycle distribution was further analyzed using a FACSCalibur flow cytometer (Becton Dickinson, San Jose, CA, USA).

### 2.11. Cell Apoptosis Analysis

The cell apoptosis assay was performed with the Annexin V-FITC/PI apoptosis kit (Dojindo, Shanghai, China). Briefly, the endometrial cells were treated with pcDNA3.1(+)-*S100A9*, pcDNA3.1(+), si-*S100A9*, and NC. The cells were collected, washed twice with pre-cooled PBS, centrifuged at 1500 rpm for 5 min, resuspended in Annexin V binding buffer, and incubated with 5 μL Annexin V-FITC and 5 μL PI solution for another 15 min at room temperature in a lightproof environment. The percentage of apoptotic cells was determined using a cell flow cytometer (Beckman Coulter, Brea, CA, USA).

### 2.12. Statistical Analysis

In this study, the data were presented as the means ± standard deviation (SD). A Student’s *t*-test was performed to evaluate the statistical analysis. *p* value < 0.05 was considered as statistically significant. * *p*< 0.05, ** *p* <0.01, and *** *p* <0.001 indicated three levels of significant differences, respectively. All experiments were three biological replicates and three technical replicates in the manuscript.

## 3. Results

### 3.1. Effects of Heat Stress on Porcine Uteri

**Figure 1:**
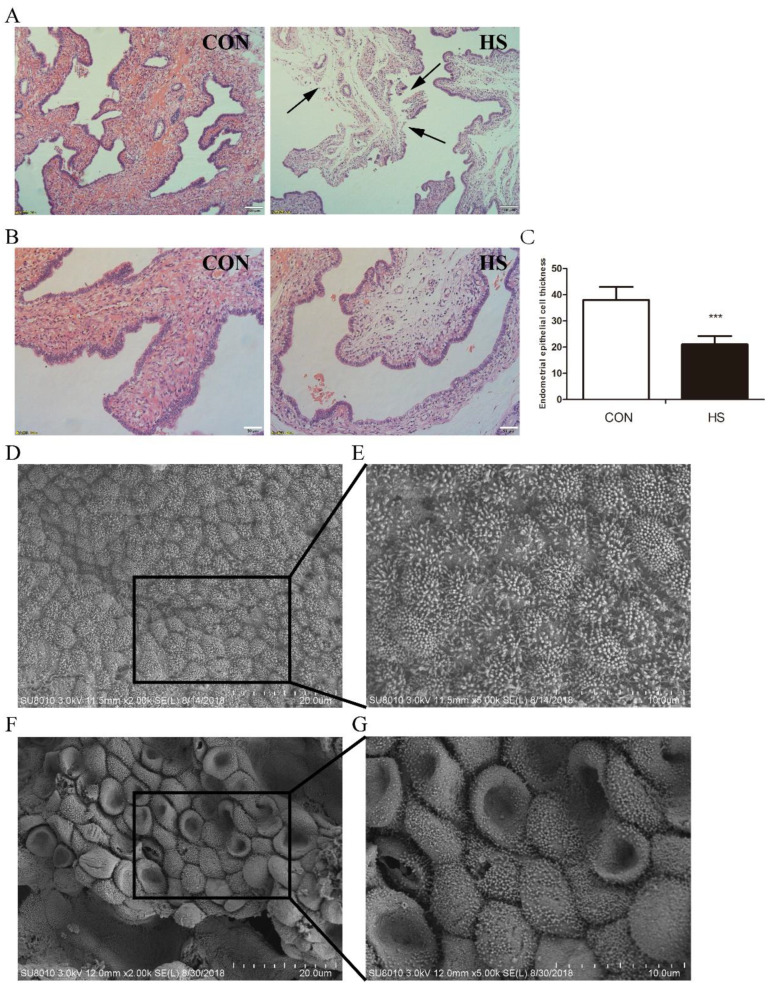
Effects of heat stress on the endometrium during embryo implantation. (**A**,**B**) Image of luminal epithelium at day 4 post heat stress (H/E staining) (scale bar, A 100 μm; B 50 μm). (**C**) Luminal epithelial thickness in the HS-treated group and the control group. *** *p* < 0.001. The data were expressed as means ± SD (*n* = 5). (**D**–**F**) Scanning electron microscopy (SEM) images of uteri under heat stress. Uteri collected on day 14 of pregnancy were treated with heat stress (scale bar, 20 μm). (**E**–**G**) High magnification (scale bar, 10 μm).

To address the pathophysiological effect of heat stress during embryo implantation, the pathological changes in the uterus were observed after hematoxylin/eosin (H&E) staining, and the epithelial thickness was analyzed. The H&E staining results showed that the luminal epitheliwum was injured, incomplete, and shed in HS-exposed porcine uteri, whereas the epithelial cells were arranged neatly and exhibited no damage in the control group (Figure 1A). Additionally, the luminal epithelial thickness (21.0613 ± 3.1871 mm) in the HS-treated group was significantly lower than that (38.0225 ± 5.0119 mm) of the control group (*p* < 0.001, *n* = 3, Figure 1B,C). Furthermore, we used scanning electron microscopy (SEM) analysis to detect the uterine ultrastructural changes under HS. The results revealed a markedly lower density of microvilli and higher cell sinking in uteri exposed to heat stress. Under HS, the uterus microvilli became much shorter and reduced, and gradually disappeared. Another intriguing change was the significantly decreased number of endometrial cells per visual field under HS (Figure 1D–G). Taken together, these results suggested that HS induced obvious uterine structure disturbances, thus provided an unfavorable environment for embryo implantation, which could eventually lead to adhesion failure of an embryo to the porcine uterine epithelial cells.

### 3.2. Heat Stress Regulated the Integrity of the Endometrium

**Figure 2:**
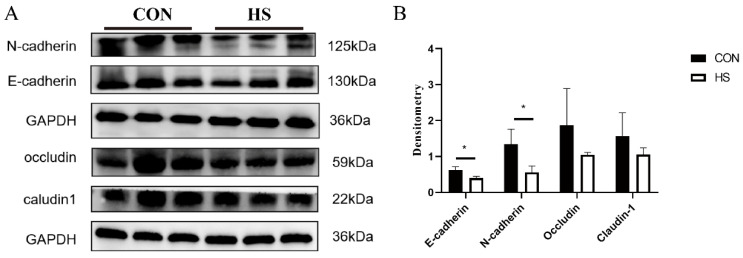
(**A**) Western blot analysis of effects of heat stress on expression levels of E-cadherin, N-cadherin, occludin, and claudin-1 proteins. (**B**) Densitometry analysis of proteins related to epithelial cell integrity. * *p* < 0.05.

Previous studies have showed that the expression and distribution of gene related epithelial integrity and junctions change during the implantation period [30]. Our results showed that the protein level of E-cadherin was significantly decreased (1.539 folds, *p* = 0.023) in the HS group, compared with that in the control group, and the N-cadherin level exhibited a similar change trend (2.382 folds, *p* = 0.040) under HS (Figure 2A). Although both occludin and claudin-1 exhibited a decrease in expression level, no statistically significant difference was observed between the HS group and the control group. These results suggested that HS impaired the barrier function of the endometrium during embryo implantation.

### 3.3. Transcriptome Analysis and DEG Identification

The total RNA was isolated from the uteri of the pigs and subjected to transcriptome sequencing. Table 1 exhibits the summary of the RNA-seq results. A total of 352,587,150 clean reads were obtained with average clean bases of 8.815 Gb per sample. The GC (guanine–cytosine) content exceeded 51.78% in all six samples. The Q30 (%) for these samples was almost above 94%. These findings indicated the reliability and validity of the RNA-seq results.

**Figure 3:**
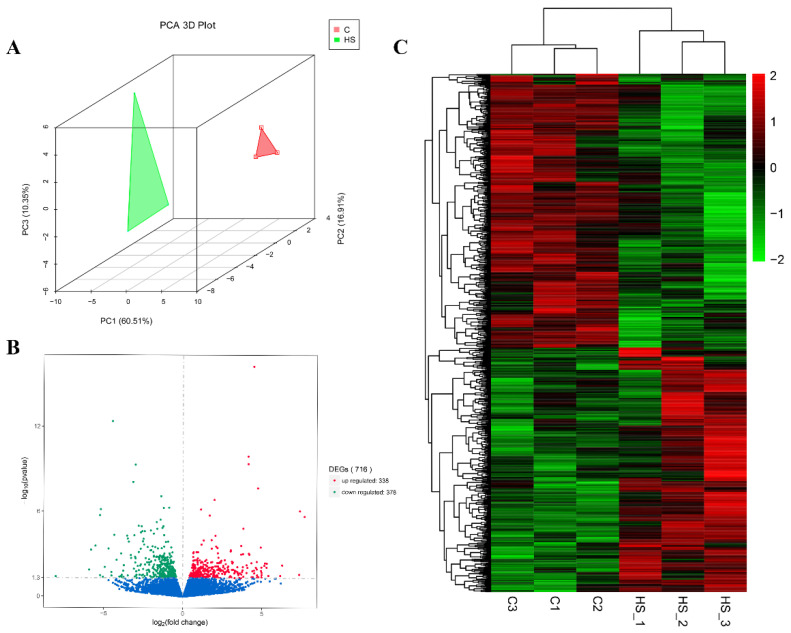
Differentially expressed genes (DEGs) analysis. (**A**) Principal component analysis (PCA) and heatmap analysis of the control and HS group samples. (**B**) Volcano map of DEGs in the control group and the HS group. (**C**) Hierarchical cluster analysis of DEGs.

The PCA (Figure 3A) and DEG clustering analyses (Figure 3C) showed that the three biological replicates were highly correlated with each other, which indicated that the samples were reproducible; thus, they could be used for subsequent experiments. A total of 716 DEGs were identified, including 338 up-regulated genes and 378 down-regulated genes (Figure 3B).

### 3.4. GO and KEGG Enrichment Analysis of DEGs

**Figure 4:**
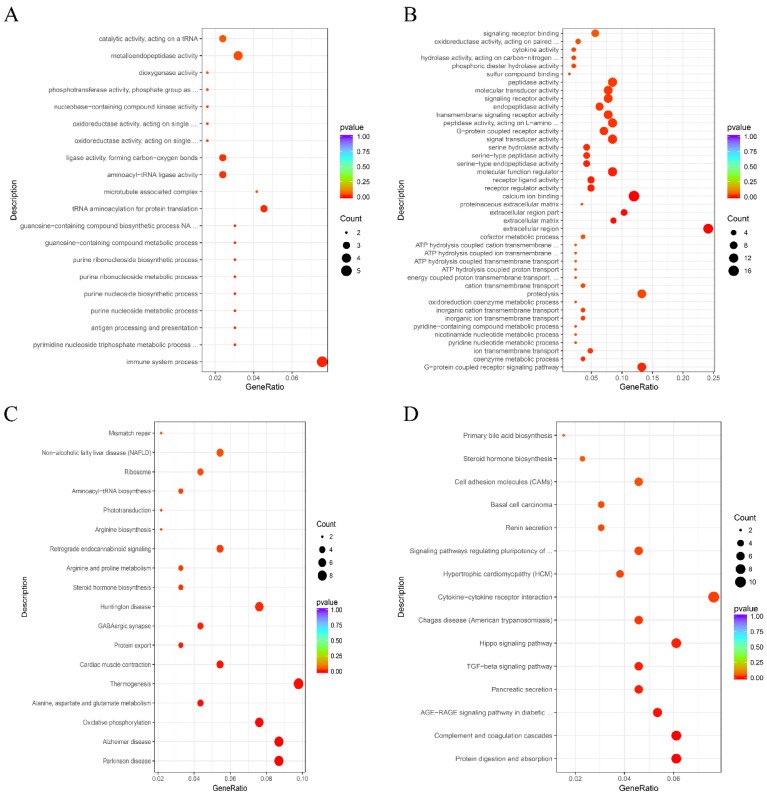
Gene ontology (GO) enrichment analysis of DEGs in the control group and the HS group during embryo implantation. (**A**) Up-regulated DEGs. (**B**) Down-regulated DEGs. Kyoto encyclopedia of genes and genomes (KEGG) analysis of DEGs in the control group and the HS group during embryo implantation. (**C**) Up-regulated DEGs. (**D**) Down-regulated DEGs.

To explore the molecular mechanism of heat stress, GO and KEGG enrichment analyses of DEGs were performed. GO analysis results indicated that the up-regulated DEGs were mainly enriched in biological processes such as the “immune system process”, “metalloendopeptidase activity”, and “aminoacyl-tRNA ligase activity”, and that the down-regulated genes were significantly enriched in binding- and signal transducer-related GO terms such as “calcium ion binding”, “extracellular region”, “molecular transducer activity”, “signal transducer activity”, and “transmembrane signaling receptor activity” (Figure 4A,B).

Furthermore, KEGG pathway analysis results showed that under heat stress, up-regulated DEGs were significantly enriched in pathways related to thermogenesis, Alzheimer disease, Parkinson disease, and the oxidative phosphorylation signaling pathway, and the down-regulated DEGs were significantly enriched in pathways related to pluripotency such as cytokine–cytokine receptor interaction, the hippo signaling pathway, the TGF-beta signaling pathway, complement and coagulation cascades, and cell adhesion molecules. Heat stress also induced the expression changes of genes regulating to the ribosome, protein export, digestion, and absorption (Figure 4C,D). All these results indicated that HS might regulate the porcine endometrium by modulating the above-mentioned pathway-related DEG expressions during embryo implantation.

**Figure 5:**
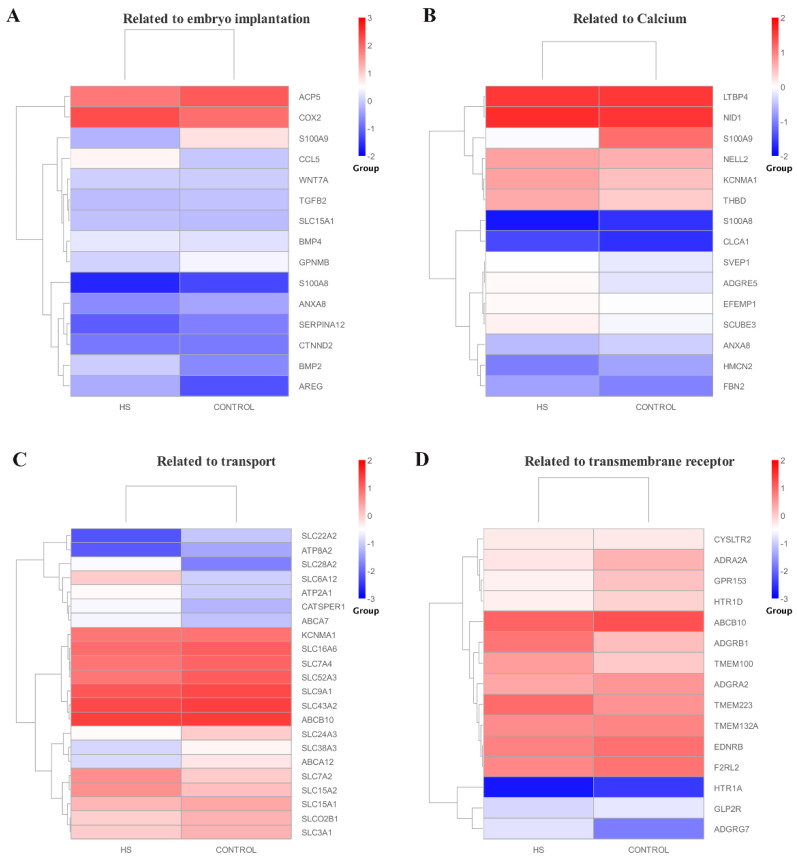
Cluster analysis of DEGs related to four major signaling pathways. (**A**) Embryo implantation. (**B**) Calcium binding. (**C**) Transport. (**D**) Transmembrane receptor. The red color presents the up-regulated, and blue color indicates the down-regulated genes.

The expression levels of four types of genes related to embryo attachment, calcium binding, transport, and transmembrane receptor were analyzed in this study, and the results showed alterations in the expression levels of these genes in the HS group. Embryo implantation-related genes such as *S100A9*, *S100A8*, and *ANXA8* were significantly reduced under HS, while *BMP2*, *CCL5*, and *COX-2* were significantly increased. The results found that most calcium binding-related genes such as *LTBP4*, *S100A8*, *S100A9*, and *SVEP1* were significantly decreased under HS (Figure 5). Collectively, these results indicated that HS exposure affected the expression levels of genes involved in endometrial calcium binding, transport, transmembrane receptor, and embryo implantation.

### 3.5. DEG Network and Validation of Real-Time PCR Results

**Figure 6:**
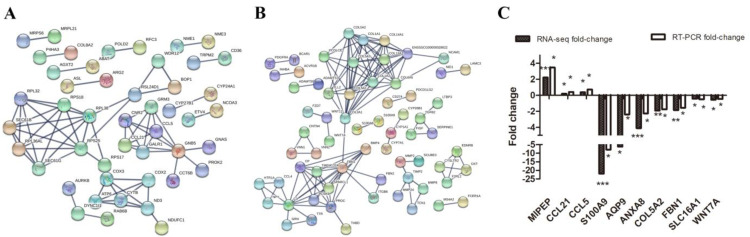
Protein–protein interaction analysis of up-regulated (**A**) and down-regulated (**B**) genes in the endometrium during embryo implantation in the HS treatment group and the control group. (**C**) Validation of the RNA-seq results by qRT-PCR. The data are presented as the means ± standard deviation (SD). * *p* < 0.05; ** *p* < 0.01; and *** *p* < 0.001.

We used the online database STRING To identify the crucial gene modules that were involved in response to heat stress among the up-regulated and down-regulated genes. Our data indicated that the HS treatment group exhibited a cluster for the genes related to ribosomal biogenesis components and a cluster for genes involved in the mitochondrion, which indicated higher transcriptional activity in the HS group than in the control group. Chemokine is a critical mediator for the migration and infiltration of immune cells [33]. In this study, chemokine gene expression was elevated after heat stress. A mitochondrion is the main place to store energy for cells, and it plays key roles in cell differentiation, information transformation, and cell apoptosis. Multiple differentially expressed genes identified in our study were involved in mitochondrion formation. The cell membrane structure-related genes include *COL1A1*, *COL5A1*, *COL5A3*, *MMP8*, and *TIMP2*, and they are the major components of the extracellular matrix and are related to extracellular matrix remodeling (Figure 6A). In this study, the down-regulation of cell membrane structure-regulated genes affected the structure of the cell membranes (Figure 6B). *S100A8* and *S100A9* are involved in anti-inflammatory system, damage repair, and the regulation of the immune response [34]. Our data showed that *S100A8* and *S100A9* played expression roles in the response to heat stress.

To verify the reliability of the RNA-seq results, a total of 10 DEGs, mainly related to embryo implantation, transmembrane, calcium, immune response, was selected for qRT-PCR (Table 2). The results showed that the expressions of *MIPEP*, *CCL21*, and *CCL5* were increased, while those of *S100A9*, *AQP9*, *ANXA8*, *COL5A2*, *FBN1*, *SLC16A1*, and *WNT7A* were decreased in response to HS. The expression profiles of these genes chosen for qRT-PCR were entirely consistent with the RNA-seq results, indicating that our RNA-seq results were reliable.

### 3.6. Effects of Heat Stress on Porcine Endometrial Epithelial Cells

**Figure 7:**
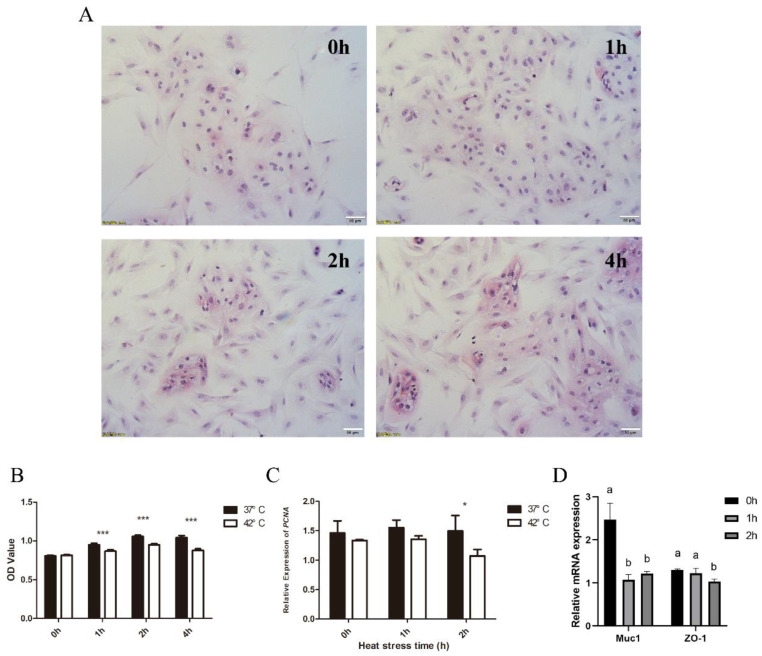
Impact of heat stress on porcine endometrial epithelial cells. (**A**) Morphological changes of EECs after heat stress. (**B**) Viability of EECs under HS. (**C**) Impact of HS on expression level of genes associated with cell proliferation (PCNA). (**D**) Impact of HS on expression level of genes associated with embryo implantation and cell adhesion (*MUC1* and *ZO-1*). * *p* < 0.05 and *** *p* < 0.001. ab means in the same bar without a common letter differ at *p* < 0.05.

To examine the effect of heat stress on porcine endometrial epithelial cells (EECs), the EECs were incubated under HS (for 1 h, 2 h, and 4 h) at 42 °C. The morphological changes of the EECs at different HS time points are shown in Figure 7. A small number of cells began to show necrosis and vacuoles in the cytoplasm at 1 h post HS. At 2 h and 4 h post HS, a small number of cells began to show necrosis, but most cells exhibited vacuoles in the cytoplasm (Figure 7A). Consistently, cell viability was significantly reduced under HS (Figure 7B). Moreover, the mRNA expression of proliferation-related *PCNA* in the EEC in the HS treatment group was remarkably lower than that in the control group (Figure 7C). *MUC1* and *ZO-1* have been reported to be related to embryo implantation and cell adhesion [35,36]. The cultured porcine endometrial epithelial cells exposed to heat stress at 42 °C for 0 h were used as a control to obtain the relative mRNA expression of *MUC1* and *ZO-1*. We wanted to investigate whether heat stress had a time-dependent effect on the expression of *MUC1* and *ZO-1*. In this study, the relative mRNA abundances of *MUC1* and *ZO-1* were significantly down regulated in response to HS, compared to those at 37 °C (Figure 7D).

### 3.7. Effect of HS on S100A9 and Determination of Its Function

**Figure 8:**
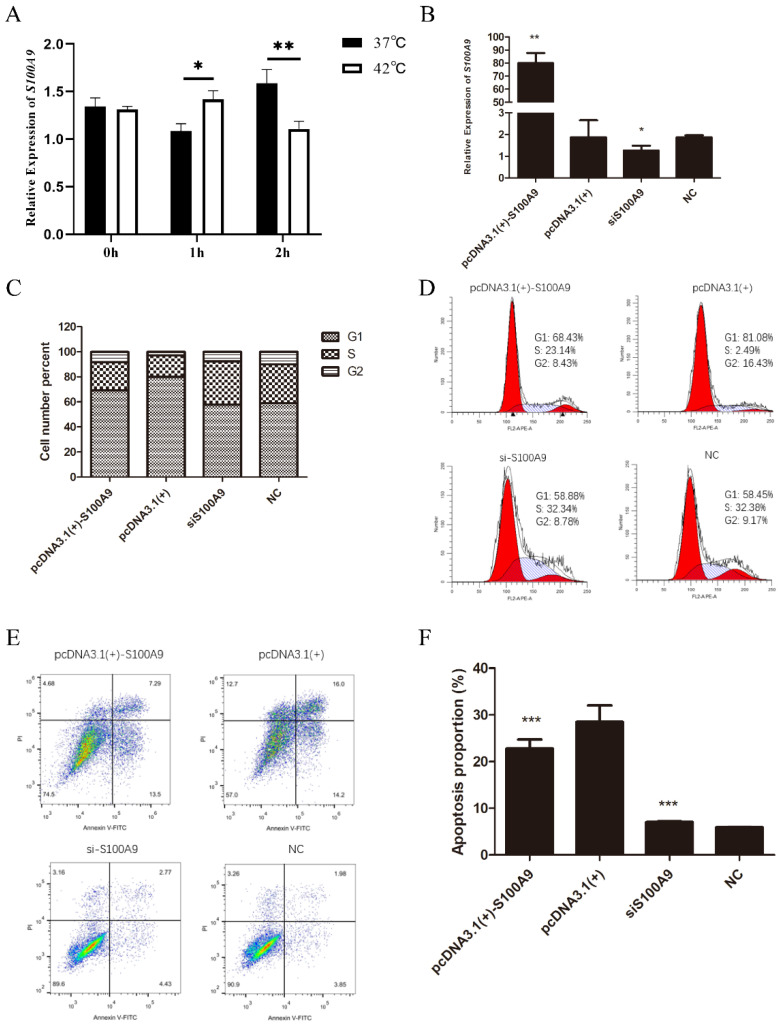
Effects of *S100A9* on proliferation and apoptosis of EECs. (**A**) Impact of HS on expression of *S100A9*. (**B**) Relative expression of *S100A9* transfected with pcDAN3.1(+)-*S100A9*, pcDNA3.1(+), siS100A9, and NC at 24 h post transfection in EECs. (**C**,**D**) Flow cytometry analysis of cell cycle distribution. (**E**,**F**) Apoptosis by flow cytometry. * *p* < 0.05, ** *p* < 0.01 and *** *p* < 0.001. G1, first gap; S, synthesis; G2, second gap; NC, negative control.

As shown in Figure 8A, the relative mRNA expression of *S100A9* was significantly decreased under HS (Figure 8A), indicating that HS might affect embryo implantation by regulating *S100A9* expression. To further explore the effect of *S100A9* on cell biological processes, we investigated the effect of *S100A9* on cell proliferation and apoptosis with a flow cytometry assay. The results of the qRT-PCR showed that *S100A9* was successfully over-expressed or inhibited (Figure 8B). The overexpression of *S100A9* resulted in a decrease from 79.94% to 69.11% in the cell number percentage in the G1 phase of cell proliferation, but an increase from 17.23% to 22.67% in the S phase (Figure 8C). Additionally, the flow cytometry analysis results showed that the expression up regulation of *S100A9* in EECs inhibited apoptosis, whereas the knockdown of *S100A9* promoted apoptosis (Figure 8D), which indicated that *S100A9* was involved in cell apoptosis and proliferation.

## 4. Discussion

Embryo implantation is complex and regulated by numerous factors in mammals. Active bidirectional interaction between an embryo and a uterus is essential for embryo adhesion during implantation. Heat stress has been reported to disrupt tight junctions in the intestine, thereby increasing intestinal permeability in mice, pigs, and humans [37,38,39]. However, the mechanism by which HS regulates the endometrium during embryo implantation remains unclear. In the present work, we focused on endometrial damage caused by HS and its potential molecular mechanisms during early embryo implantation. The uterine environment is an important factor contributing to successful embryo implantation. Endometrial thickness has long been considered as a very important indicator of the uterine environment [40]. Our results showed that the structures of the uteri were different between the control group and the HS group, and that HS could induce endometrial defects, making its structure incomplete during the implantation window stage. Notably, the microvilli became fewer and shorter in the HS group. HS inhibited endometrial epithelial cell proliferation in pigs and reduced the expression of endometrial receptivity-related MUC1 and ZO-1. Moreover, the endometrium thickness was significantly reduced in the HS group. Our data indicated that HS induced an endometrium disorder, resulting in an adverse maternal microenvironment for embryo implantation.

Cytoskeleton structures are critical for maintaining the morphology and mechanical properties of cells. Therefore, it is of great significance to unravel the influence of HS on the cytoskeleton. Tight junctions have been confirmed to be essential in porcine early pregnancy. Tight junction-related E-cadherin plays an important role in maintaining the morphological and structural integrity and polarity of epithelial cells [30]. However, the effect of HS on paracellular permeability remains unclear, especially in the endometrium. In the present study, we found that under HS, the endometrium exhibited defective tight junctions. N-cadherin and E-cadherin are crucial for the adhesion and invasion of the blastocyst. Our results indicated that their expression levels in the endometrium were significantly lower in the HS group than in the control group. Furthermore, ITSN1 is a cellular bridging protein involved in biological processes such as endocytosis, cytokinesis, cell signaling, cell survival, and actin cytoskeleton rearrangement [41]. As an important regulator of microtubule dynamics, *MAP2* regulates the assembly of microtubule proteins [42]. Our data indicated that the expressions of cytoskeletal-associated genes were down-regulated, such as *ITSN1*, *MAP2*, and *ARHGAP33*. These results further indicated that HS could damage the integrity of the endometrial cells and change their permeability through the down regulation of these genes, thus preventing the adhesion between the embryo and endometrial cells.

To further explore the corresponding molecular mechanisms, we performed RNA-seq analysis to identify the genes and pathways in response to HS. The RNA-seq showed that 338 genes were significantly up regulated and 378 genes were remarkably down regulated between the two groups (HS vs. control). HS was also found to decrease the expression of calcium adhesion-related CAM and undermine the balance between ions and amino acid transporters.

Calcium ions are key regulators for both embryo implantation and microvilli formation, and they can provide a greater surface area on the apical side of the cells; thus allowing asymmetric localization of membrane transporters, cytoskeletal proteins, and enzymes on EECs, eventually enhancing cell polarity [35]. Previous studies have revealed that blocking endometrial calcium channels can cause complete implantation failure in mice during the implantation window stage [43]. Calcium ions are also involved in nutrient and other molecule transport. In pigs, the expression of calcium-encoding molecules, such as integrins, *SPP1*, *S100A9*, and *S100A8*, in the endometrium are significantly up regulated during conceptus implantation [44]. In a previous study, *SLC24A3*, which is possibly involved in reproduction function during the estrous cycle in female rats, was reported to be abundantly expressed in the uterus, and its expression was up regulated by E2 and P4 [45]. Previous studies have shown that heat stress leads to a decrease in Ca^2+^-ATPase activity, thus causing a calcium imbalance in the sarcoplasmic reticulum [46]. In a present study, the maintenance of calcium in the endometrial cells seemed to play a pivotal role in response to HS, which was supported by the significant change in the expression of genes associated with calcium homeostasis, calcium transport, and inflammation, such as *ANXA8*, *LTBP4*, *S100A8*, *S100A9*, *CLCA1*, *ATP2A1*, and *SLC24A3*. Our data also showed that HS caused significant down regulation of calcium binding-related *ANXA8* and *LTBP4*. As a member of the membrane-linked protein family, *ANXA8* has been reported to enhance the activity of the sarcoplasmic reticulum Ca^2+^-ATPases and express a high value on day 12 of pregnancy in pigs [47]. *LTBP4* is an essential protein for the formation and maturation of microfibrils [48]. In our study, the up regulation of these two proteins might promote calcium uptake and increase intracellular free calcium ion content, which has the potential to alleviate the disturbance of calcium ion homeostasis caused by heat stress. As calcium effectors, *S100A8* and *S100A9* have been previously found to exhibit a specific and high expression pattern in porcine endometria at day 12 post implantation [44]. Concurrent evidence has indicated that in *S100A8^(−/−)^* mice, embryonic development failed [49]. This current study found that the expression of *S100A9* and *S100A8* was decreased 95-fold and 87-fold, respectively, indicating that these two genes were key regulators impacting uterus function in high temperatures. A previous study revealed that extracellular *S100A9* causes proliferation, migration, and invasion of HP75, which was consistent with our results that *S100A9* could promote proliferation and suppress apoptosis in endometrial cells [50]. The low expression of *S100A8* and *S100A9* might prevent further progression of the implantation process and embryonic growth. However, the action mechanism of calcium in the endometrium remains to be further examined. These results further indicated that the destruction of endometrium homeostasis might be responsible for microvillus abnormity induced by HS.

Embryo implantation is a structural connection that is established based on the interaction between an embryo and the maternal endometrium, which allows material exchange, and it plays an essential role in mammalian reproduction. The expression change of amino acid transporters in the endometrium is closely related to membrane lesions. For example, amino acid transporters and their metabolism in the endometrium are associated with the receptive state of the endometrial tissue at both day 4 and day 7 of pregnancy [51]. Previous studies have shown that maternal HS during early gestation to mid-gestation in pigs decreased the expression of genes associated with the placental amino acid, peptide, and cation transmembrane transport [52]. As a derivative of this amino acid, *SLC16A1* is important for fetal and placental development, and its expression was decreased upon hyperosmotic stress [53]. *SLC15A2* expression was down regulated in EID (embryo implantation dysfunction) mice, and this gene might be useful to repair an injury after an intrauterine perfusion treatment [54]. Our GO enrichment analysis showed that the mRNA expression of transport-related genes including *SLC24A3*, *SLC22A2*, *SLC38A3, SLC52A3*, *SLC15A1*, *SLC16A6*, *SLC43A2*, *SLC9A1*, *SLC7A4*, *SLC3A1*, and *SLC16A1* were up regulated, but that of other transport-related genes including *SLC28A2*, *SLC6A12, SLC15A2*, *SLC7A2*, and *SLC26A8* were down regulated under HS.

## 5. Conclusions

**Figure 9:**
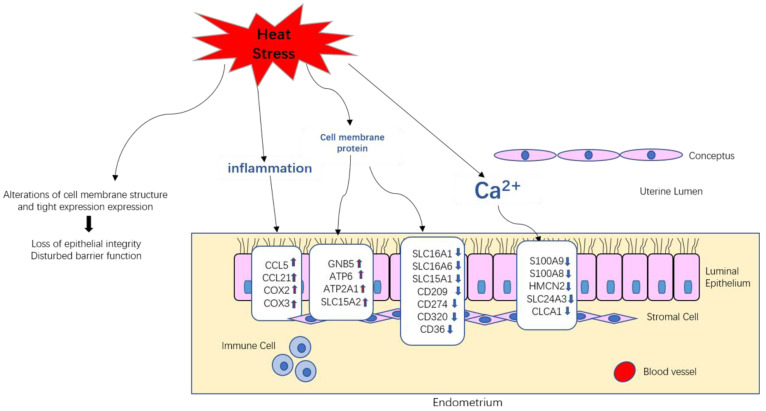
Model of heat-stress effects on endometria in pigs during embryo implantation.

In summary, the present work demonstrated that heat stress could lead to uterine structural damage, which was supported by histopathology, cell viability, cell proliferation, and cell integrity investigations (Figure 9). HS suppresses the expressions of cell adhesion-related genes including N-cadherin, E-cadherin, MUC1, and ZO-1. A variety of DEGs related to calcium binding and amino acid transport have been identified, of which the most significant, differentially expressed, *S100A9* was regulated by HS in the uterus. Our results provided a new perspective from calcium ions for understanding unsuccessful implantation in porcine endometrium. Our findings also offered a novel insight into the response of a maternal uterus to HS during the implantation process. One limitation of our study was that there were only three biological repeats. Despite this issue, our results were still true and reliable.

## Figures and Tables

**Table 1:** Summary of the RNA-seq results of porcine uterine RNA samples.

Sample	Raw Reads	Clean Reads	Clean Bases	Error Rate	Q30	GC pct
C1	53,298,596	52,575,622 (98.64%)	7.89 G	0.02	94.62	52.96
C2	66,589,734	65,620,474 (98.54%)	9.84 G	0.03	94.07	52.74
C3	53,313,686	52,579,282 (98.62%)	7.89 G	0.02	94.34	52.48
HS_1	64,540,558	63,720,642 (98.72%)	9.56 G	0.02	94.43	51.78
HS_2	65,701,188	64,863,356 (98.72%)	9.73 G	0.03	94.28	52.8
HS_3	53,889,090	53,227,774 (98.77%)	7.98 G	0.03	94.13	52.57

Notes: C, control group; HS, heat stress group; error rate: the overall sequencing error rate of the data; Q30: the percentage of bases with Phred values greater than 30 in total bases; GC pct: the percentage of G (guanine) and C (cytosine) in the clean reads.

**Table 2:** Primers for sequences.

Gene Symbol	Primers Sequences	Size (bp)	Tm (℃)
*ANXA8*	F: CTTCTGAGTGCAGCAGGGG	183	63.3
	R: GTCGGGGTCTGGGTTGAAG		
*AQP9*	F: CAGTCGCGGACATTTTGGAG	127	59.0
	R: CAAGGCAAAAGACACGGCTG		
*CCL5*	F: CACACCCTGCTGTTTTTCC	151	58.0
	R: CCATTTCTTCTCTGGGTTGG		
*CCL21*	F: TGGCTCAGTCACTGGTTCTG	144	60.0
	R: GGTAGCTGCGTACAACGTGA		
*COL5A2*	F: TAGTGCTGAAAGAAGAGCCCG	128	61.3
	R: GTCTTGCTTCTGCCCAGTTTG		
*FBN1*	F: ACCGGAGATGGCTTCACTTG	235	61.3
	R: TCTCACACTCACAGCGGAAC		
*MIPEP*	F: CCACGGAGATGGCTTCACTTG	114	60.0
	R: CCCGCGACATCAGGTATGAG		
*S100A9*	F: TCCTGGGCTTGGACAGAGT	133	61.3
	R: CTTTCTGGTTCAGGGTGTCCC		
*SLC16A1*	F: GCATGGGCATCAACTACCGA	166	61.3
	R: TTGGGGCTTCCTTCTATGCC		
*WNT7A*	F: TCTTGCCCTCAGCATCACAG	219	58.0
	R: ACAGGCTTTGTCCACACCTC		
*PCNA*	F: ATGCCTTCTGGTGAATTTGC	116	58.0
	R: TTTCCGAGTTCTCCACTTGC		
*MUC1*	F: GGGCTTCTGGGACTCTTT	143	56.0
	R: AGGTTATAGGTGCCTGCTT		
*ZO-1*	F: GCCATCCACTCCTGCCTAT	111	59.7
	R: GGGACCTGCTCATAACTTCG		
*β-Actin*	F: GCCTCACTGTCCACCTTCCA	184	59.0
	R: AGCCATGCCAATGTTGTCTCTT		

## Data Availability

The datasets generated for this study can be found in the NCBI SRA (SUB10962240).
